# 伴11号染色体长臂异常的儿童成熟B细胞淋巴瘤11例临床研究

**DOI:** 10.3760/cma.j.issn.0253-2727.2023.11.007

**Published:** 2023-11

**Authors:** 楠 张, 彦龙 段, 春菊 周, 玲 金, 菁 杨, 爽 黄, 梦 张, 楠 李

**Affiliations:** 1 国家儿童医学中心，首都医科大学附属北京儿童医院儿童肿瘤中心肿瘤内科，儿童血液病与肿瘤分子分型北京市重点实验室，儿科重大疾病研究教育部重点实验室，北京 100045 Medical Oncology Department, Pediatric Oncology Center,Beijing Children's Hospital, Capital Medical University, National Center for Children's Health,Beijing Key Laboratory of Pediatric Hematology Oncology, Key Laboratory of Major Diseases in Children,Ministry of Education, Beijing 100045, China; 2 国家儿童医学中心，首都医科大学附属北京儿童医院病理科，北京 100045 Medical Oncology Department, Pediatric Oncology Center,Beijing Children's Hospital, Capital Medical University, National Center for Children's Health,Department of Pathology

**Keywords:** B细胞淋巴瘤, 11号染色体, 儿童, 临床, 治疗, B-Cell Lymphoma, Chromosome 11, Children, Clinical, Treatment

## Abstract

**目的:**

探讨伴11号染色体长臂（11q）异常的儿童成熟B细胞淋巴瘤（MBCL）的临床特征及预后。

**方法:**

回顾性分析2018年12月至2023年2月首都医科大学附属北京儿童医院收治的11例伴11q异常的MBCL患儿的临床资料。

**结果:**

11例儿童MBCL患者中男9例，女2例，中位年龄9（2～13）岁，中位病程1.8（0.5～24）个月。临床表现为颈部淋巴结肿大4例，鼻塞、打鼾4例，腹部疼痛2例，呼吸困难1例。病理形态呈伯基特淋巴瘤样7例，滤泡性淋巴瘤样2例，高级别B细胞淋巴瘤样2例。所有患者均无中枢神经系统、骨髓累及，B超及PET/CT等影像学评估未见广泛转移，1例有巨大瘤灶。修订国际儿童非霍奇金淋巴瘤分期系统（IPNHLSS）Ⅱ期4例，Ⅲ期5例，Ⅳ期2例。11q探针检测显示，5例11q增益，3例11q缺失，3例增益和缺失同时存在。FISH显示3例患者C-MYC基因阳性，伴11q异常的高级别B细胞淋巴瘤8例，伴11q异常的伯基特淋巴瘤3例。根据国家卫生健康委员会2019版儿童侵袭性成熟B细胞淋巴瘤诊疗规范，A组化疗1例、B组2例、C组8例，早期评估疗效均完全缓解；B组及C组于中期评估后降低化疗强度，2例化疗中，其余9例中位随访32（6～45）个月均无事件生存。

**结论:**

伴11q异常的儿童MBCL发病率低，临床症状轻、进展慢，行FISH检测存在11q异常，无MYC、BCL2、BCL6重排为其重要诊断要点，降低化疗强度预后良好。

## Abstract (en)

**Objective:**

To explore the clinical, pathological, diagnostic, treatment, and prognostic features of children with mature B-cell lymphoma (MBCL).

**Methods:**

This retrospective study included pediatric patients with MBCL with chromosome 11 long-arm abnormalities who were diagnosed and treated at our hospital from December 2018 to February 2023.

**Results:**

Among the 11 pediatric patients with MBCL, nine were male and two were female, with a median age of 9 (2−13) years and a median disease course of 1.8 (0.5−24) months. The clinical manifestations were cervical lymph node enlargement in four patients, nasal congestion and snoring in four patients, abdominal pain in two patients, and difficulty breathing in one patient. There were seven cases of Burkitt's lymphoma, two of follicular lymphoma, and two of advanced B-cell lymphoma according to the pathological morphology examination. No patients had central nervous system or bone marrow involvement, and no extensive metastasis was observed on B-ultrasound or positron emission tomography-computed tomography (PET/CT). One patient had a huge tumor lesion. The Revised International Pediatric Non-Hodgkin Lymphoma Staging System classified four patients as stage Ⅱ, five as stage Ⅲ, and two as stage Ⅳ. 11q probe detection showed five cases of 11q gain, three of 11q loss, and three of both gain and loss. FISH showed positive MYC expression in three patients, including eight with advanced B-cell lymphoma with 11q abnormalities and three with Burkitt's lymphoma with 11q abnormalities. According to the 2019 edition of the National Health Commission's diagnostic and treatment guidelines for invasive MBCL in children, one patient was classified as Group A, two as Group B, and eight as Group C. Early evaluation of the efficacy showed complete remission. After mid-term evaluation, the intensity of chemotherapy was reduced in Group B and Group C. Among two cases of chemotherapy, the remaining nine cases had a median follow-up of 32 (6−45) months, and none had event-related survival.

**Conclusion:**

The incidence of MBCL with 11q abnormalities in children is low, clinical symptoms are mild, and progression is slow. The absence of MYC, BCL2, BCL6 rearrangements, C-MYC negative and 11q abnormalities on FISH is an important diagnostic indicator, and reducing the intensity of chemotherapy can improve prognosis.

2017年版WHO淋巴组织分类将伴11号染色体长臂（11q）异常的伯基特样淋巴瘤认定为新的诊断亚型，因为与伯基特淋巴瘤（BL）有着相似的病理形态和免疫表型，其既往被认为是BL的一部分。但由于其缺乏C-MYC基因的重排，具有特征性的11q改变，且随着国内外报道的增多，11q异常还可以表现于滤泡淋巴瘤（FL）、高级别B细胞淋巴瘤（HGBCL），因此在2022年WHO淋巴瘤组织分类中将其更名为伴11q异常的HGBCL（HGBCL-11q）。此外研究报道11q可以同时出现在C-MYC阳性的成熟B细胞淋巴瘤（MBCL）中[1]。本文对北京儿童医院诊断及治疗的11例伴11q异常的MBCL患儿进行回顾性总结，进一步探讨其临床特征、病理特征及分子遗传学特点。

## 病例与方法

1. 病例：本研究纳入2018年12月至2023年2月于北京儿童医院行FISH检测伴11q异常的MBCL患儿11例，所有患儿家长均签署书面知情同意书。

2. 组织病理及免疫组化检查：所有标本均行苏木精-伊红染色（HE）和免疫组织化学染色；免疫组织化学染色采用Envision两步法，并设立阴性、阳性对照。FISH检测的位点为11q22.3、11q23.3、11q24.3、11q25（探针均购于广州安必平医疗科技有限公司，探针具体信息如下：11q22.3基因探针，代码：F.01035-01，探针名称：GSP ATM/CSP11；11q23.3/11q24.3基因缺失探针，代码：F.01315-01，探针名称：GSP 11q23.3/GSP 11q24.3；11q25基因缺失探针，代码F.01386-01，探针名称GSP D11S1037/CSP11）；C-MYC、BCL2、BCL6基因检测，检查结果交由首都医科大学附属北京友谊医院、北京大学第三医院及北京儿童医院3家医院病理科会诊，得出诊断。病理诊断标准依据2022年WHO淋巴造血系统肿瘤修订版第5版，临床分期按照国际儿童非霍奇金淋巴瘤分期系统（IPNHLSS）根据肿瘤侵犯范围分为Ⅰ～Ⅳ期。

3. 治疗方案：化疗方案按照国家卫生健康委员会2019版儿童侵袭性成熟B细胞淋巴瘤诊疗规范，根据不同分期及危险因素分为 A组（2个疗程）、B组（6个疗程）、C组（10个疗程）3组治疗方案，予分层化疗，总疗程2～6个月。根据化疗早、中、后期评估瘤灶缩小及残留情况调整化疗方案：B组于第3轮将COPADM方案（包括长春新碱、醋酸泼尼松、甲氨蝶呤、环磷酰胺、柔红霉素）中环磷酰胺用药3 d减量至2 d，C组于第二轮将CYVE方案（包括阿糖胞苷、依托泊苷）中大剂量阿糖胞苷由每天3 g/m^2^减量至每天2 g/m^2^。A组及B组不应用利妥昔单抗；C组中，免疫功能正常者应用利妥昔单抗，剂量为375 mg/m^2^，每周1次，总疗程4次。

4. 随访：通过门诊进行随访，全部治疗结束后两年内每3个月行一般性评估，除临床体检外还包括瘤灶相关超声、免疫功能、肝功能、乳酸脱氢酶；两年内每3个月评估1次，两年后每6个月评估1次，在一般评估基础上，行甲状腺、心脏等发育及脏器功能评估。随访时间截至2023年2月1日。

5. 统计学处理：应用SPSS 26.0软件进行统计描述，计量资料呈正态分布以*x*±*s*表示，呈偏态分布以中位数（范围）表示。

## 结果

1. 一般情况：符合本文纳入标准的共11例患儿，占同期北京儿童医院收治侵袭性成熟B细胞淋巴瘤的4.4％（11/248），男9例，女2例；发病年龄2～13岁。确诊前病程0.5～24个月，其中4例以鼻塞、打鼾起病，4例以发现颈部包块起病，2例以腹痛起病，1例以呼吸困难起病；受累部位局限于头颈部5例，肠道2例，多部位受累4例。其中8例为HGBCL-11q，3例为伴11q异常的伯基特淋巴瘤（BL-11q），起病至随访期间均未发现转移。临床分期Ⅱ期4例，Ⅲ期5例，Ⅳ期2例。

2. 组织病理学表现：对11例患儿进行回顾性病理复习，其中8例为HGBCL-11q，3例为BL-11q。8例HGBCL-11q患儿中，光镜下例2、例8为FL样结构，为大的、可扩张的、高度增生的滤泡样结构，肿瘤性滤泡互相融合，可见显著星空现象，具有突出的幼稚细胞样形态（图1）；例4、例6为HGBCL样结构，肿瘤细胞中等大小，形态大小一致，细胞核圆形，核分裂象明显；其余4例为BL样结构，细胞中等偏小，细胞核呈现圆形，嗜碱性细胞质，可见明显的核分裂象、核碎片及星空现象。免疫组化均表达CD20（图2A）、CD10、BCL6（图2B）；8例免疫组化C-MYC均阴性；Ki-67阳性指数90％～95％（图2C），不表达BCL2；4例检测LMO2均为阳性；4例检测CD38均为阴性。8例患儿FISH均未检测出BCL6、BCL2的重排（表1）。FISH应用11q探针检测提示5例存在11q的增益；例1、例6、例7存在11q22.3增益，例4、例5存在11q23.3增益；例2存在11q22.3缺失（图3），例3同时存在11q22.3的增益及11q23.3、11q24.3、11q25的缺失（表1）。

**图1:**
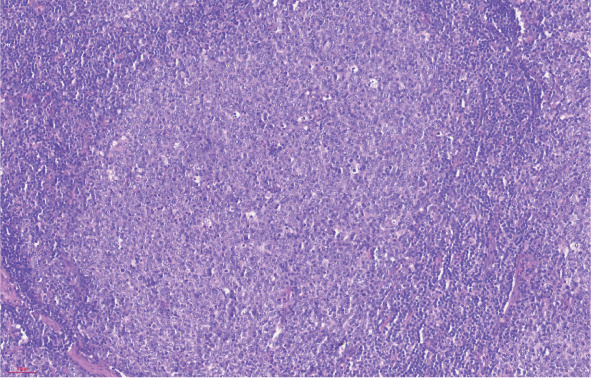
伴11q异常的高级别B细胞淋巴瘤肿瘤组织光镜下呈滤泡淋巴瘤样结构（HE染色，×100）

**图2:**
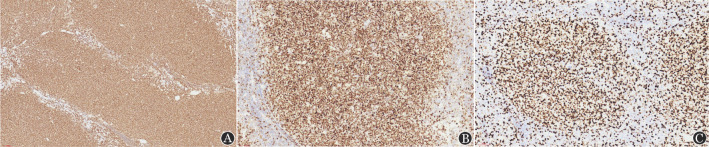
伴11q异常的高级别B细胞淋巴瘤免疫组化染色结果（Envision法） A CD20阳性，×100； B BCL6阳性，×200； C Ki-67阳性指数>90％，×200

**图3:**
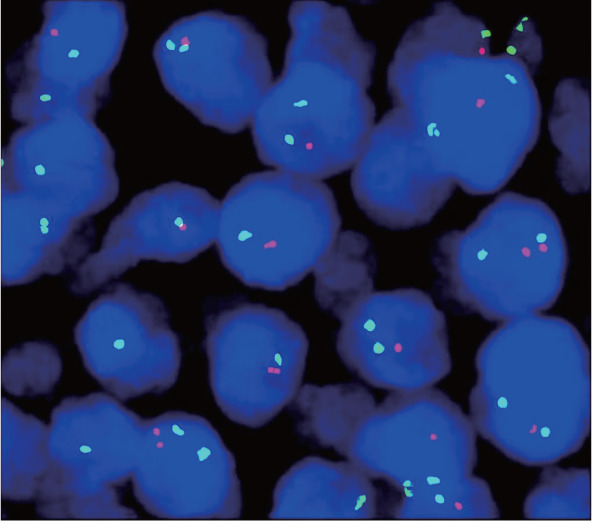
伴11q异常的高级别B细胞淋巴瘤FISH法应用11q22.3探针检测结果 注 红色荧光标记目标11q22.3区段；绿色荧光标记内参CSP11区段；可见较多1红2绿信号类型细胞，提示11q22.3基因区段缺失

**表1:** 8例伴11q异常的高级别B细胞淋巴瘤（HGBCL）患儿的临床特征、病理特征及分子遗传学特点

例号	性别	年龄（岁）	临床表现	起病时间（月）	受累部位	分期	病理形态	免疫组化				FISH检测		
								BCL6	C-MYC蛋白	LMO2	CD38	C-MYC基因	11q增益	11q缺失
1	男	7	颈部包块	1	颈部右侧，左肾，扁桃体右侧，胸椎	Ⅲ期	BL	+	−	+	−	−	+	−
2	女	2	腹痛、血便	2	回肠及系膜周围淋巴结，颈部双侧、腋窝、腹股沟淋巴结	Ⅲ期	FL	+	−	+	−	−	−	+
3	男	8	颈部包块	2	舌根、口咽、悬雍垂、会厌	Ⅳ期	BL	+	−	NT	NT	−	+	+
4	女	10	颈部包块	3	颈部右侧淋巴结	Ⅱ期	HGBCL	+	−	NT	NT	−	+	−
5	男	6	腹痛	0.5	回盲部肠管，腹腔右侧、腹膜后及肠系膜	Ⅲ期	BL	+	−	NT	NT	−	+	−
6	男	13	打鼾	0.5	咽扁桃体，颈部右侧及颌下淋巴结	Ⅱ期	HGBCL	+	−	+	−	−	+	−
7	男	9	鼻塞	1.5	鼻咽部	Ⅱ期	BL	+	−	+	−	−	+	−
8	男	13	颈部包块	24	淋巴结（右腮腺、颈部双侧、纵隔、双肺门、腹腔、腹膜后、腹股沟右侧）、脾脏，鼻咽	Ⅲ期	FL	+	−	NT	NT	−	−	+

注 BL：伯基特淋巴瘤；FL：滤泡淋巴瘤；NT：未检测；+：阳性；−：阴性

3例BL-11q的患儿，免疫组化均表达CD20、CD10、BCL6、C-MYC、CD38；不表达BCL2、LMO2；3例患儿 FISH均未检测出BCL6、BCL2的重排。FISH应用11q探针检测提示例1同时存在11q22.3的增益及11q23.3、11q24.3、11q25的缺失；例2存在11q23.3增益及11q24.3缺失；例3存在11q24.3的缺失（表2）。

**表2:** 3例伴11q异常的伯基特淋巴瘤（BL）患儿的临床特征、病理特征及分子遗传学特点

例号	性别	年龄（岁）	临床表现	起病时间	受累部位	分期	病理形态	免疫组化				FISH检测		
								BCL6	C-MYC	LMO2	CD38	C-MYC基因	11q增益	11q缺失
1	男	12	打鼾	2个月	双侧扁桃体	Ⅱ期	BL	+	+	−	+	+	+	+
2	男	9	鼻塞、打鼾	3个月	鼻咽	Ⅲ期	BL	+	+	−	+	+	+	+
3	男	11	呼吸困难	1周	鼻咽、腮腺、颈部淋巴结、睾丸、脑膜	Ⅳ期	BL	+	+	−	+	+	−	+

注 +：阳性；−：阴性

3. 治疗及预后：11例化疗分组中A组1例，B组2例，C组 8例，化疗前免疫功能均正常，C组均应用利妥昔单抗，中期评估均完全缓解，故B组及C组予以减轻化疗强度；目前2例正化疗中，其余9例化疗后随访肿瘤均完全缓解，中位随访32（6～45）个月，均无事件生存。

## 讨论

HGBCL-11q是2022年WHO淋巴组织分类提出的一个新的淋巴瘤亚型，临床罕见，目前对于儿童HGBCL-11q归纳总结性研究较少，本文总结既往国内外儿童伴11q异常的HGBCL检索到文献共8篇[1]–[8]（54例），其中男性38例（70％），起病年龄为11.5（2～17）岁；可获得瘤灶受累部位的临床资料的有52例，以结内为主（38例，73％），结外病变以咽淋巴环为主（29例），34例报告了临床分期，以Ⅰ～Ⅲ期为主（31例，94％）。既往研究报道认为与BL相比，HGBCL-11q病史短、临床分期早、侵袭性弱、更容易累及到淋巴结[7],[9]。本研究中HGBCL-11q共8例，其中男性病例较多（6/8），与既往报道相符，中位年龄为8.5（2～13）岁，较既往报道年龄更小，受累部位头颈部及腹部淋巴结3例（3/8），结外5例（腹部1例，咽淋巴环1例，多部位受累3例），与既往报道不相符。研究病例病程短，临床分期以Ⅱ～Ⅲ期为主（7/8），与既往报道相符。同时研究报道11q可出现在移植后和免疫功能低下的BL中[10]，本文BL-11q 3例，均为男性，均以鼻咽部肿物起病，1例伴有结外受累（睾丸、腮腺等），尿酸、乳酸脱氢酶等肿瘤溶解指标正常，与传统的BL相比，范围局限，侵袭性弱，发生肿瘤溶解的概率小。

病理和免疫组化方面，HGBCL-11q通常与BL相似，但病理形态上HGBCL-11q巨噬细胞和凋亡小体的数量减少[7],[11]，并且可呈现出HGBCL、FL的病理形态[5],[7]。免疫组化与BL的不同点是：MYC蛋白的表达水平较BL低（<50％）或不表达[4],[9]，LMO2、CD16、CD56高表达（>50％），缺乏CD38、CD45的表达[4],[11]，国内外学者认为免疫组化的表达水平可作为BL-11q的重要诊断要点。本研究报告的8例HGBCL-11q中，4例为BL样的形态，另外4例中2例病理形态为FL样，2例病理形态分类为HGBCL，体现了细胞学的多形性。免疫组化方面，除了表达BL的表型外，均不表达MYC蛋白，与既往报道相符，因本文为回顾性研究，有些病例未进行LMO2、CD56、CD38、CD45的检测；4例检测LMO2的患儿中均为阳性，4例检测CD38的患儿中均为阴性，支持既往报道。本文中3例BL-11q均符合BL的形态，无巨噬细胞和凋亡小体的减少，免疫组化MYC蛋白的高表达（>70％），CD38、CD45高表达（>50％），未检测出LMO2、CD16、CD56的表达[4]。结果支持LMO2、CD16、CD56的高表达，CD38、CD45的表达减少作为HGBCL-11q的重要诊断要点的观点。

分子遗传学方面，HGBCL-11q行FISH检测中无MYC、BCL2、BCL6基因的断裂，同时存在11q的异常，其异常可表现为增益、缺失或者增益及缺失同时存在。11q23的增益在侵袭性B细胞淋巴瘤中多见，但11q24的缺失并不多见，可作为HGBCL-11q和其他类型的鉴别[4]。除了特征性的11q异常，HGBCL-11q在染色体及基因变异方面，HGBCL-11q更接近于HGBCL或DLBCL[6]–[8]。相关研究认为染色体畸变的不同导致了其调控基因的不同，导致肿瘤特征与BL不同，如11q23.2-q23.3区域的增益，可引起PAFAH1B2的过表达；11q24的缺失可以通过调控FLI1、ETS1、NFRKB，导致肿瘤的发生[6]。另有对HGBCL-11q的研究表明，microRNA hsa-mir-34b通路的下调可能是替代MYC易位的致病机制[12]。因此认为HGBCL-11q在分子水平上是一个不同于BL的淋巴瘤类型[8]，进而导致临床进展及预后的不同。

诊断方面，HGBCL-11q的诊断可分为两部分：①病理形态及免疫组化符合HGBCL-11q的特征；②当形态和病理表型呈现BL样时，首先行免疫组化检测C-MYC蛋白的阳性率，如果大于80％阳性，倾向诊断BL，如果C-MYC阳性率<80％，可行FISH检测MYC、BCL2、BCL6的重排，如果只有MYC重排阳性，则可诊断BL，如果三者均阴性则应进行11q染色体的检测，呈现特异的11q区域异常，则诊断具HGBCL-11q[9]。此外在MYC阳性的BL及MYC阳性的HGBCL中也可出现11q的异常[13]–[14]，因此缺乏MYC重排为HGBCL-11q诊断的重要标准[2]。本研究中，3例患儿免疫组化提示C-MYC蛋白呈强阳性，且FISH可检测出C-MYC、11q重排，故诊断为伴有BL-11q，8例C-MYC蛋白阴性，且FISH检测出11q阳性，MYC、BCL2、BCL6的重排阴性，故诊断HGBCL-11q明确。

治疗及预后方面，国内外学者报道的可获得临床资料的43例患者中，均予以化疗，化疗方案为HyperCAVD方案、R-CHOP方案、NHL-BFM5等，其中15例联合利妥昔单抗治疗，均完全缓解，随访时间（49±12）个月，均无事件发生，学者认为在未来的治疗过程中可以酌情降低化疗强度[5]。本文中的11例11q阳性的MBCL患儿，予以国家卫生健康委员会2019版儿童侵袭性成熟B细胞淋巴瘤方案联合或不联合利妥昔单抗化疗，中期评估后均完全缓解，因考虑此病侵袭性弱，B组方案于第3轮COPADM方案将环磷酰胺减量至2 d，C组于第二轮CYVE方案中大剂量阿糖胞苷由3 g/m^2^减量至2 g/m^2^，均取得良好的预后，其中2例BL-11q正处于化疗中，化疗过程中未出现肿瘤溶解，本文中患儿较其他侵袭性MBCL化疗过程中发生事件少，预后好，提示11q异常的MBCL有更好的预后，可降低化疗强度。

本文将MBCL-11q分为HGBCL-11q和BL-11q进行讨论，HGBCL-11q多发生于儿童及青少年，起病病灶局限，病情进展慢，侵袭性弱，病理形态可呈现BL样、FL样、HGBCL样，在免疫组化方面，除了B细胞表型外，LMO2、CD16、CD56的高表达，CD38、CD45的表达减少可作为其重要诊断要点。分子生物学的诊断要点为HGBCL-11q不存在MYC、BCL2、BCL6的重排，降低化疗强度可取得良好的预后。同时本文中3例BL-11q较其他侵袭性淋巴瘤病灶局限、侵袭性弱，减低化疗强度后疗效好。以上均提示未来对MBCL-11q的治疗可尝试降低化疗强度。
